# Effects of Bicarbonate Ions in Tea Brewing Water on Sensory Properties and Substances of Different Teas

**DOI:** 10.3390/foods15111958

**Published:** 2026-06-02

**Authors:** Wanjun Gao, Xing Liang, Lanying Li, Yu Yao, Bo Zhang, Yongjiang Hu, Fan Luo, Dongna Liu

**Affiliations:** 1 Tea Research Institute, Tea Resources Utilization and Quality Testing Key Laboratory of Sichuan Province, Sichuan Academy of Agricultural Sciences, Chengdu 610066, China; lxing@scsaas.cn (X.L.); lly187@scsaas.cn (L.L.); yaoyu@scsaas.cn (Y.Y.); luofan@scsaas.cn (F.L.); 2 Sichuan Yihuo Quanjiang Beverage Co., Ltd., Guangyuan 628000, China; zhangbo@eho-life.com (B.Z.); huyongjiang@eho-life.com (Y.H.)

**Keywords:** tea, brewing water, sensory properties, HCO_3_^−^

## Abstract

This study investigated the effect of formula water based on soda water and HCO_3_^−^ on the sensory evaluation and substances of different teas. Highly fermented teas showed better sensory quality with a higher soda water ratio in the formula, while low-fermented teas improved with a small amount of soda water. Low HCO_3_^−^ (≤21.97 mg/L) enhanced aroma and reduced bitterness, whereas high HCO_3_^−^ (≥40.27 mg/L) can dim the brightness of tea infusion, dull the aroma, and reduce refreshing taste. High HCO_3_^−^ is unfavorable to green tea’s substance stability but somewhat beneficial to that of black tea. When brewing tea with TBW (tea brewing water), a longer brewing time yields more substances dissolved by HCO_3_^−^. Compared to four waters, TBW was better for green and black teas. In short, multiple brews with TBW were best. This study provides a reference for the optimization of the quality of tea brewing water.

## 1. Introduction

Tea, as a beverage with a long history, is loved by people for its rich cultural connotations, diverse sensory flavors, and natural health attributes [1,2]. Without water, tea cannot be made, and without good water, tea cannot be fully appreciated [2,3]. Tea and water combine to form a tea infusion, and water has a greater impact on the tea infusion than tea. Water directly affects the quality of the tea infusion [4,5]. The formation of the color, aroma, and taste of tea infusion is closely related to the complex chemical components in tea leaves, and the water quality of brewed tea is one of the key factors in this chemical change [6]. Some studies focusing on the ions in water have proved that the content and composition of ions in water will affect the quality of tea infusion. The current research on the influence of ions in water on the quality of tea infusion mainly focuses on cations. However, there is relatively little research on anions, which also play an important role in the quality of tea infusion [5,7].

The bicarbonate ion (HCO_3_^−^) is one of the common ions in natural water, which has a significant impact on water quality and plays an important role in stimulating the sensory quality of tea color, aroma, and taste [8]. HCO_3_^−^ keeps the tea infusion in a stable, weakly alkaline environment, which ultimately leads to the oxidation and decomposition of tea polyphenols (such as catechins), reducing the purity of the tea aroma and thus affecting the overall flavor quality of the tea [7,9]. HCO_3_^−^ is an alkaline ion in water, which can neutralize acidic substances in water and act as a buffer for pH. As the concentration of HCO_3_^−^ in water increases, the pH of the water gradually increases, and the water quality exhibits weak alkalinity or alkalinity [10]. Among them, when the concentration of HCO_3_^−^ in the water is 120.82 mg/L, the pH value of the water is stable within the range of 8.15 ± 0.01. In addition, the dissociation behavior of HCO_3_^−^ directly affects the conductivity of water. In water, HCO_3_^−^ can dissociate into hydrogen ions (H^+^) and carbonate ions (CO_3_^2−^), increasing the electrolyte content in water [11,12]. Studies have shown that pH and conductivity are important factors affecting the quality and stability of tea infusion and tea beverages [13,14]. Therefore, it is vital to choose the appropriate water during the process of brewing tea.

Many factors contribute to the sensory quality of tea [15]. The flavor components in tea are very complex, and changes in type, content, and proportion will affect the taste of the tea infusion [16]. Of course, the growth environment of tea trees, the processing technology of tea leaves, and even external factors such as brewing conditions and brewing equipment also affect the taste of tea leaves. The aroma and taste perceived by people refer to the comprehensive expression of water-soluble substances in tea infusion after stimulating the human sense of smell and taste [17]. The taste of tea infusion is characterized by five types of flavorings: bitter, astringent, sour, sweet, and fresh [18]. It is presented by the water-extracted substances in tea infusion, which are mainly composed of tea polyphenols, amino acids, caffeine, water-soluble pectin, soluble sugars, soluble proteins, water-soluble pigments, vitamins, and inorganic salts [19,20]. Among them, water-soluble tea polyphenols, amino acids, caffeine, soluble sugars, etc., account for 30% to 48% of the tea water extract substances [21].

This article systematically studied the effects of different proportions of soda water on the sensory evaluation of seven types of tea. The regulatory effect of HCO_3_^−^ concentration changes in tea brewing water on the sensory quality of tea was studied. At the same time, the influence of different brewing times on the water-soluble substances of green tea and black tea was studied in order to provide new scientific insights for enhancing the quality of brewing water, tea leaves, and tea beverages.

## 2. Materials and Methods

### 2.1. Chemicals and Reagents

The tea samples are commercially available green tea (special grade Maofeng), yellow tea (Mengding Huangya), white tea (Baimudan), oolong tea (special grade Tieguanyin), black tea (Sichuan Kung Fu (bud tea)), dark tea (4 g pressed tea cake), and scented tea (Piaoxue). The appearance of the tea samples is shown in Figure S1.

The water sample for brewing tea is made up of soda water and raw water provided by Sichuan Yihuo Quanjiang Beverage Co., Ltd. (Guangyuan, China) in the proportions of S1 (1:3), S2 (1:5), S3 (1:8), S4 (1:10), S5 (1:12), S6 (1:15), S7 (1:20), S8 (1:30)s and S9 (1:40). The control is purified water (CK).

NaHCO_3_ was added to water to prepare tea brewing water containing 7 types of different concentrations of HCO_3_^−^. The concentrations of HCO_3_^−^ were SY1 120.82 mg/L, SY2 40.27 mg/L, SY3 21.97 mg/L, SY4 15.10 mg/L, SY5 11.50 mg/L, SY6 7.80 mg/L, SY7 5.89 mg/L. The control was deionized water (CK).

Folin phenol, gallic acid, pH 8.0 phosphate-buffered solution, and L-theanine were purchased from Beijing Solarbio Science & Technology Co., Ltd. (Beijing, China). Sodium carbonate, indenone, and stannous chloride were purchased from Chengdu Cologne Chemical Co., Ltd. (Chengdu, China). EDTA-2Na and ascorbic acid were purchased from China National Pharmaceutical Group Chemical Reagent Co., Ltd. (Shanghai, China). Acetonitrile, acetic acid, and methanol were all HPLC grade and from Xilong Science Co., Ltd. (Shantou, China). Authentic standards were purchased from Chengdu Efa Biotechnology Co., Ltd. (Chengdu, China), including caffeine, gallic acid, (+)-Catechin (C), (−)-epicatechin (EC), (−)-gallocatechin (GC), (−)-epigallocatechin (EGC), (−)-catechin gallate (CG), (−)-epicatechin gallate (ECG), (−)-gallocatechin gallate (GCG), and (−)-epigallocatechin gallate (EGCG). Ultrapure water (18.2 MΩ) was obtained using a Cascada III.I5 Water Purification System (Pall Corporation, Show Low, AZ, USA) and used to prepare all the solutions.

### 2.2. Sensory Evaluation

In the absence of a Human Ethics Committee at the institution, we ensured that the sensory evaluation followed stringent protocols to protect the rights and privacy of all participants. These measures included securing voluntary participation, providing comprehensive information about the study’s requirements and potential risks, obtaining explicit written or verbal consent from participants, maintaining the confidentiality of participant data, and allowing participants the freedom to withdraw from the study at any time.

Five professional assessors within the age range of 23 to 30 years (2 males, 3 females) evaluated samples. Samples weighing 3.0 g were infused in white porcelain cups with 150 mL of freshly boiled water for 4 min. A comprehensive sensory quality evaluation refers to the national standard (GB/T 23776-2018) [22] tea evaluation method. The total sensory score was evaluated by quality scores using a 100-point scale, with 40% for the aroma, 20% for the liquor color, and 40% for the taste. Samples were assessed three times through blind evaluation [23].

### 2.3. Optimization of Brewing Time

Samples weighing 3.0 g were infused in white porcelain cups with 150 mL of freshly boiled water for 1 min, and then the infused liquid was taken out for testing. Then, 150 mL of water was added and allowed to brew for 1 min before the tea infusion was taken out for testing. According to the above method, samples with brewing times of 1, 2, 3, 5, 7, and 10 min were obtained in sequence.

### 2.4. Verification Test

Compared with tea brewing water for green tea (TBW1) and black tea (TBW2), purified water (PW), tap water (TW), and mineral water (MW) were selected as controls for validation experiments.

### 2.5. Measurement of Chemical Components in Samples

The water extract refers to the national standard (GB/T 8305-2013) [24]. The contents of tea polyphenols in tea refer to the national standard (GB/T 8313-2018) [25], and the content of free amino acids in tea refers to the national standard (GB/T 8314-2013) [26]. Gallic acid (GA), catechin (C), epicatechin (EC), epigallocatechin (EGC), epicatechin gallate (ECG), gallocatechin gallate (GCG), epigallocatechin gallate (EGCG), catechin gallate (CG), and caffeine (CAF) contents of the extracts were determined using high-performance liquid chromatography (HPLC). The sample solution and detection method were prepared as described in GB/T 8313-2018. Briefly, 0.2 g of tea powder was extracted with 5.0 mL 70% (*v*/*v*) methanol by stirring at 70 °C for 10 min, and then centrifuged at 3500 r/min for 10 min at room temperature. Thereafter, the supernatant was collected, and the extraction was repeated once. The obtained supernatants were mixed together to make a volume of up to 10.0 mL with 70% (*v*/*v*) methanol and filtered through a 0.45 μm Millipore filter. The extracts were analyzed using a 1260 Infinity II HPLC system (Agilent Technologies Inc., Palo Alto, CA, USA) equipped with an ultraviolet detector. Chromatographic separation was performed on a 1260 Infinity II liquid chromatography C18 column (Phenomenex 250 mm × 4.6 mm, 5 μm), with a flow rate of 1.0 mL/min at 35 °C. Optical density was determined at 278 nm, and three replications were performed for each experiment.

## 3. Results and Discussion

### 3.1. pH Value and Conductivity of Different Formula Water and Its Sensory Evaluation on Tea

The pH values and conductivity of tea infusion with different formula waters are shown in Table 1. The pH value of the brewed tea infusion with the formulation with a ratio of 1:3 is stable at 8.12 ± 0.00, and the conductivity is 252.0 μs/cm.

The sensory evaluation results of seven kinds of tea brewed with nine formula waters, shown in Table 2 and Table S1, demonstrated that there were significant differences in the sensory evaluation of the different teas brewed with different formula waters. The sensory evaluation score of water sample S8 was the highest after brewing green tea (94.60 ± 0.45). Under this water sample, the green tea infusion color was bright yellowish green, the chestnut aroma was rich and lasting, and the taste was fresh and heavy, with a brisk and sweet aftertaste (Table 2 and Figure S2). The sensory evaluation score of water sample S7 was the highest after brewing white tea (93.20 ± 0.37) and scented tea (93.50 ± 0.45). Under this water sample, the white tea infusion color was bright apricot yellow, the tip aroma was rich and lasting, and the taste was sweet and fresh, as well as brisk. The scented tea under this water sample had a bright light greenish yellow soup color, with a flowery aroma that was fresh, lovely, rich, and lasting, and the taste was fresh and heavy, with a brisk and sweet aftertaste. The sensory evaluation score of water sample S6 was the highest after brewing black tea (94.67 ± 0.50), yellow tea (95.10 ± 0.70), and oolong tea (93.87 ± 0.46). Under this water sample, the black tea infusion color was bright red, and the sweet aroma was rich and lasting; the taste was fresh and sweet, as well as brisk. The yellow tea infusion had a bright honey yellow color; a tender, sweet, flowery aroma; and the taste was heavy and mellow, sweet and brisk. The oolong tea infusion color was bright light yellowish green, the clean and flowery aroma was rich, and the taste was heavy and mellow, smooth and brisk. The sensory evaluation score of water sample S4 was the highest after brewing dark tea (95.00 ± 0.28). Under this water sample, the dark tea had a bright and rich red soup color; the aroma after aging was pure and normal, rich and lasting; and the taste was mellow and thick, with a sweet aftertaste and smooth, having an aged flavor. According to the processing methods, tea can be divided into unfermented, semi-fermented, and fermented tea [27]. The results showed that the fermentation degree was positively correlated with the proportion of soda water in the formula water, and when this correlation held, the sensory quality of tea was the best. Fermented teas typically contain fewer reactive catechins and more polymerized polyphenols, which respond differently to alkalinity. The main component of soda water is HCO_3_^−^. The reason that affects the sensory quality of tea may be the different concentration of HCO_3_^−^ [9], resulting in different alkalinity levels in the tea infusion.

### 3.2. The Influence of HCO_3_^−^ on the Sensory Evaluation of Different Types of Tea

The different brewing waters had a significant impact on the sensory evaluation of tea. HCO_3_^−^ is one of the important ions in water; it can directly affect the pH and conductivity of water (Table S2) and the sensory quality of tea. As such, we further investigated the effects of the seven different concentrations of HCO_3_^−^ on the sensory quality and physicochemical properties of different types of tea.

Sensory evaluation of different teas was conducted using seven water samples containing different concentrations of HCO_3_^−^. The results, shown in Table S3, indicate that the sensory evaluation differences between different water samples were significant. For green tea, white tea, oolong tea, and scented tea, SY5 (HCO_3_^−^ concentration of 11.50 mg/L) had the highest sensory scores of 93.87 ± 0.29, 93.20 ± 0.43, 93.07 ± 0.05, and 94.37 ± 0.21, respectively. Compared with CK, the sensory score of SY5 significantly increased (*p* < 0.05), mainly manifesting as an increase in the material sensation of the green tea infusion, an increase in the freshness of the aroma, a slight increase in the bitterness of the taste, but a better coordination for green tea; a richer aroma and better freshness and sweetness in taste for white tea; increased intensity and persistence of aroma and a richer and smoother taste for oolong tea; and increased freshness and richness of aroma, and a richer and more refreshing taste for scented tea. For black tea and yellow tea, the sensory scores of SY4 (HCO_3_^−^ concentration of 15.10 mg/L) were the highest, at 94.43 ± 0.24 and 92.60 ± 0.33, respectively. A significant difference was observed with CK (*p* < 0.05), mainly manifesting as a richer, sweet aroma and a sweeter and more mellow taste for black tea; and a richer and higher aroma, and a more refreshing and harmonious taste for yellow tea. For dark tea, the sensory score of SY3 (HCO_3_^−^ concentration of 21.97 mg/L) was the highest, at 94.07 ± 0.41, which is significantly different from CK and the other water samples (*p* < 0.05), mainly manifesting in a richer and more persistent aged aroma, and a smoother and richer taste.

The sensory evaluation results show that the presence of low concentrations of HCO_3_^−^ (≤21.97 mg/L) was beneficial for enhancing the fresh and brisk taste and reducing the bitter and astringent taste. This may be related to the pH changes in tea infusion caused by HCO_3_^−^, as well as the leaching and reaction of biochemical components during tea infusion [9,28]. Low concentrations of HCO_3_^−^ may stimulate the release of tea aroma compounds, while neutralizing some acidic substances in the tea infusion, which helps to reduce bitterness and endows the tea infusion with a fresh and harmonious taste. On the other hand, high concentrations of HCO_3_^−^ (≥40.27 mg/L) can lead to a decrease in the brightness and color of tea infusion, a dull aroma, a reduced taste, and a decrease in refreshing taste. Similar studies have found that HCO_3_^−^ had a significant negative correlation with aroma acceptability for tea infusion [9]. The results indicated that high concentrations of HCO_3_^−^ have a strong acidity buffering ability, which stabilizes tea infusion in a weakly alkaline environment. This may cause changes in the leaching rate of pigments, polyphenols, and other substances within the tea, intensify oxidation reactions, and consequently result in a darkening of tea infusion color and a reduced refreshing taste [5]. In addition, the results of this study found that the responses of different tea types to the concentration of HCO_3_^−^ in brewed tea water are not consistent. Tea types with low fermentation levels may be more suitable for brewing with water containing low concentrations of HCO_3_^−^ [14], while tea types with high fermentation levels can be brewed with water having a relatively high concentration of HCO_3_^−^.

### 3.3. Effects of Different Concentrations of HCO_3_^−^ on Substances in Tea Infusion

**Figure 1:**
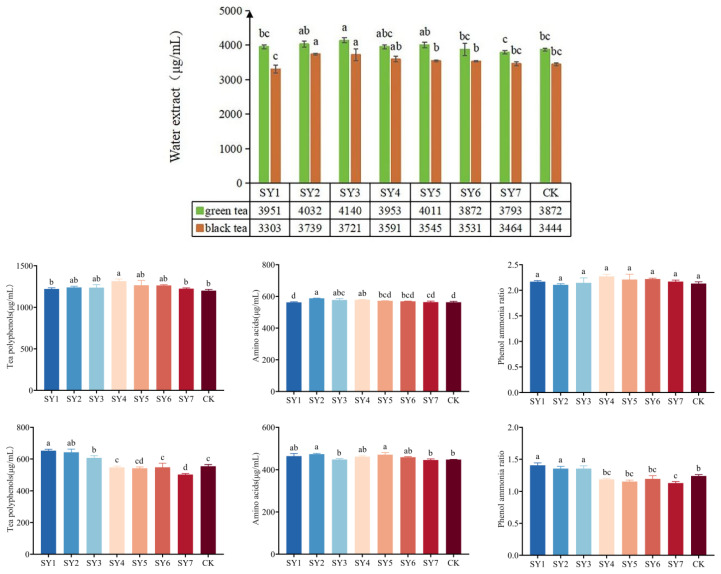
The effect of different concentrations of HCO_3_^−^ in brewing water on the total amount of water extract, tea polyphenols, amino acids, and phenol ammonia ratio in green tea and black tea. Note: Significant differences between the eight brewing water samples (including CK) are indicated by different lowercase letters (*p* < 0.05).

In the experiment, in order to study the effect of different concentrations of HCO_3_^−^ on the substances in tea infusion, green tea (non-fermented) and black tea (fermented) were selected and brewed with water samples of different concentrations of HCO_3_^−^. Then, the total amount of tea water extract, polyphenols, and amino acids was measured, and the phenol ammonia ratio was calculated (Figure 1). Compared with CK, brewing with SY3 significantly increased the total amount of water extract of green tea by 6.9%, and brewing black tea with SY2 and SY3 significantly increased this amount by 8.6% and 8.0%, respectively. HCO_3_^−^ had a certain effect on the dissolution of tea polyphenols and amino acids in green tea, but had no significant effect on the phenol ammonia ratio. After brewing with SY1-CK, the content of tea polyphenols and amino acids increased at first and then decreased, and the highest content of tea polyphenols was 1315.57 μg/mL (SY4), that of amino acids was 588.24 μg/mL (SY2), and compared with CK, it increased by 9.55% and 4.41%, respectively, in green tea infusion. Compared with CK, SY1–SY3 (≥9.29%) significantly enhanced the dissolution of tea polyphenols in black tea. SY2 and SY5 significantly increased the content of amino acids in black tea infusion by 5.73% and 5.21%, respectively. SY1, SY2, and SY3 significantly increased the phenol ammonia ratio by 13.44%, 9.30%, and 9.30%, respectively, while that of SY7 significantly decreased by 8.94%.

Simultaneously detect the content of catechins and caffeine (Table S4). In green tea samples, compared to CK, SY1–SY7 had no significant effect on EC, EGCG, ECG, and CG in green tea infusion, the contents of GC, C, GA, and CAF were the lowest in SY3, SY1, SY4, and SY4, respectively, and were significantly reduced by 27.9%, 79.7%, 32.2%, and 6.6%, respectively. But after brewing with SY7, the EGC content significantly increased by 8.3%. In black tea samples, compared with CK, the highest content of GC, C, CG, GA, and CAF increased by 15.9% (SY5), 125.4% (SY1), 12.9% (SY3), 9.3% (SY4), and 19.5% (SY7), respectively. In addition to ECG, the content of C, CG, GA, and CAF in tea infusion showed an increasing trend after brewing from CK to a high concentration HCO_3_^−^ solution. The results of the effects of different concentrations of HCO_3_^−^ on the substances in tea infusion show that a high concentration of HCO_3_^−^ is not conducive to the stability of substances in green tea infusion, but it is beneficial to black tea infusion to a certain extent. Ions in brewing water may affect the existence of substances in tea infusion, and HCO_3_^−^ can affect the pH in tea infusion, making some substances oxidize faster, and may participate in the reaction of multiple substances, so as to change the content of substances in tea infusion [5].

### 3.4. The Effect of Different Brewing Times on the Content

According to the sensory evaluation of different teas brewed with formula water and HCO_3_^−^ aqueous solution, and the effect of HCO_3_^−^ aqueous solution on the substances in green tea and black tea infusion, the results showed that formula water S8 was the best for brewing green tea (as a tea brewing water, hereafter named TBW1) and formula water S6 was the best for brewing black tea (TBW2). Further experiments were conducted to investigate the effects of different brewing times on the water extracts, catechins, and caffeine of green tea and black tea when brewing with TBW1 and TBW2, respectively.

**Figure 2:**
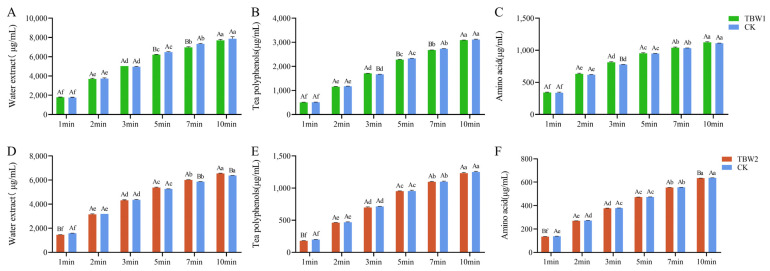
The effects of different brewing times on water extract, tea polyphenols, and amino acids in green tea (**A**–**C**) and black tea (**D**–**F**) soup. Note: Lowercase letters represent the significance analysis of different brewing times under the same water sample; the significance analysis of different water samples under the same brewing time is indicated using capital letters.

According to Figure 2, the water extract, tea polyphenols, and amino acids of green tea and black tea both significantly increased when brewed with TBW1 and TBW2 at different brewing times, respectively. Compared with CK, the water extract and tea polyphenols in green tea samples were significantly reduced when brewing for 5 min and 7 min. However, tea polyphenols and amino acids increased significantly when brewing for 3 min. After brewing with TBW1, the contents of catechins and caffeine increased significantly with the increase in brewing time (Table 3). There was no significant difference in most catechins (EGC, C, EC, CG, and GA) and caffeine between TBW1 and CK at the same brewing time. Compared with CK, the water extract in black tea samples increased significantly when brewing with TBW2 for 7 min and 10 min, while the water extract, tea polyphenols, and amino acids decreased significantly when brewing for 1 min. GC, C, CG, and CAF increased initially and then tended to be stable with the increase in brewing time when brewing with TBW2 (Table S5). C, CG, and GA contents were significantly lower than those in CK when brewed with TBW2 for 1–10 min. The results showed that when brewing tea with TBW, the longer the brewing time, the more substances in the tea were dissolved under the action of HCO_3_^−^, which also accompanied the intensification of oxidation reactions; therefore, the oxidation rate in green tea infusion was faster. HCO_3_^−^ had the greatest impact on catechins and had no effect on caffeine. Therefore, it can be brewed multiple times in a short period of time to optimize quality when brewing tea with TBW.

**Figure 3:**
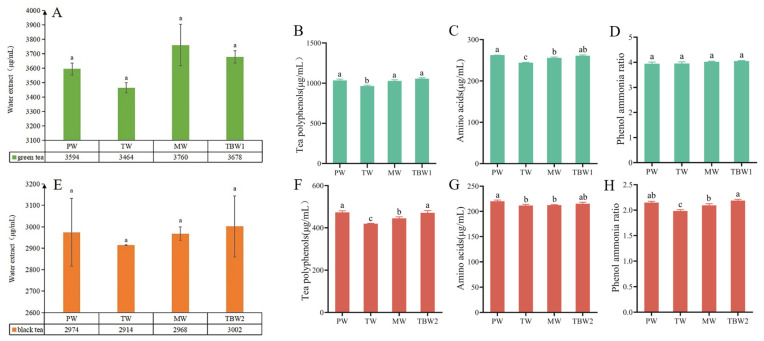
The effects of 4 kinds of water on water extract, tea polyphenols, amino acids, and phenol ammonia ratio in tea infusion after brewing green tea (**A**–**D**) and black tea (**E**–**H**). Note: Significant differences between the four waters are indicated by different lowercase letters (*p* < 0.05).

In order to understand the effects of TBW and three types of water commonly used for brewing tea in daily life on the substances in non-fermented (green tea) and fermented (black tea) tea infusion, the tea brewing water for green tea (TBW1) and black tea (TBW2), purified water (PW), tap water (TW), and mineral water (MW) were selected to carry out the experiment. In Figure 3, the results showed that there was no significant difference in the water extract (green tea and black tea) and phenol ammonia ratio (green tea) across the four types of water; the contents of tea polyphenols (green tea and black tea), amino acids (green tea), and phenol ammonia ratio (black tea) in tea infusion brewed with TW were significantly lower than those in the other three kinds of water. The content of EGC, C, EC, EGCG, ECG, CG, and caffeine in green tea infusion brewed with TBW1 was the highest (Table S6), which were 132.41 μg/mL, 66.56 μg/mL, 282.32 μg/mL, 439.46 μg/mL, 85.28 μg/mL, 62.90 μg/mL, and 411.90 μg/mL, respectively. Across all water types, the phenol ammonia ratio in black tea infusion brewed with TBW2 was the highest. These results indicate that TBW is a more suitable choice for brewing green tea (TBW1) and black tea (TBW2) among the four kinds of water.

## 4. Conclusions

In this study, different formulations of water based on soda water were used to study their effects on the sensory evaluation of different teas, as well as the effects of HCO_3_^−^, the most important component of soda water, on the sensory evaluation and substances of different teas. The results indicated that teas with high fermentation levels, such as black tea and dark tea, have better sensory quality when brewed with formula water containing a higher proportion of soda water. However, we found that brewing low fermented tea, such as green tea, white tea, and scented tea, with formula water containing a small amount of soda water would significantly improve their sensory quality. The best brewing water for fresh and refreshing tea samples was around S8, while the best brewing water for sweet and mellow tea samples was around S6. Low concentrations of HCO_3_^−^ (≤21.97 mg/L) may stimulate the release of tea aroma compounds, while neutralizing some acidic substances in the tea infusion, which helps to reduce bitterness and endows the tea infusion with a fresh and harmonious taste. On the other hand, high concentrations of HCO_3_^−^ (≥40.27 mg/L) can lead to a decrease in the brightness and color of tea infusion, a dull aroma, a reduced taste, and a decrease in refreshing taste. The high concentration of HCO_3_^−^ is not conducive to the stability of substances in green tea infusion, but it is beneficial to black tea infusion to a certain extent. HCO_3_^−^ can affect the pH in tea infusion, make some substances oxidize faster, and may participate in the reaction of multiple substances, so as to change the content of substances in tea infusion. In addition, when brewing tea with TBW, the longer the brewing time, the more substances in the tea were dissolved under the action of HCO_3_^−^, which also accompanied the intensification of oxidation reactions, and the oxidation rate in green tea infusion was faster. HCO_3_^−^ has the greatest impact on catechins and has no effect on caffeine. Compared across four kinds of water, TBW was a more suitable choice for brewing green tea (TBW1) and black tea (TBW2). Therefore, it can be brewed multiple times in a short period of time to achieve the best brewing effect when brewing tea with TBW. However, the concentration of HCO_3_^−^ in the water used for brewing tea exerts a complex and significant influence on the color, aroma, and taste of the tea infusion, which demands specific optimization in accordance with the different types and grades of tea. This study emphasizes the importance of taking into account the characteristics of different tea types when selecting brewing water; it provides a reference for the optimization of the quality of brewing water, as well as strategies for potential industrial applications, e.g., bottled tea, specialty brewing water, and beverage formulation.

## Figures and Tables

**Table 1:** Physicochemical properties of formula water.

Sample	Concentration of HCO_3_^−^ (mg/L)	Concentration of Na^+^ (mg/L)	pH	Conductivity (μS/cm)
S1	82.75	42.78	8.12 ± 0.00	252.0
S2	55.17	28.52	7.89 ± 0.01	112.3
S3	36.78	19.01	7.83 ± 0.00	83.1
S4	30.09	15.55	7.68 ± 0.00	75.2
S5	25.46	13.16	7.57 ± 0.00	69.0
S6	20.69	10.69	7.54 ± 0.00	65.1
S7	15.76	8.15	7.42 ± 0.00	47.2
S8	10.68	5.52	7.34 ± 0.02	30.3
S9	8.07	4.17	7.21 ± 0.01	18.2

**Table 2:** Sensory evaluation of green tea and black tea after brewing with formula water.

Tea	Water	Soup Color (20%)		Aroma (40%)		Taste (40%)		Total Score
		Comment	Score	Comment	Score	Comment	Score	
Green tea	S1	greenish yellow; approaching bright	89.50 ± 0.71	chestnut aroma, more stuffy	89.33 ± 0.47	heavy and mellow; more fresh, sweet aftertaste	89.33 ± 1.25	89.37 ± 0.73 e
	S2	greenish yellow; approaching bright	90.83 ± 0.85	chestnut aroma, more stuffy	90.67 ± 0.94	heavy and mellow; more fresh, sweet aftertaste	90.17 ± 0.62	90.50 ± 0.62 d
	S3	greenish yellow; more bright	93.33 ± 0.85	chestnut aroma, more stuffy	92.00 ± 0.82	heavy and mellow; more fresh, sweet aftertaste	91.67 ± 0.47	92.13 ± 0.29 c
	S4	bright greenish yellow	94.00 ± 0.82	chestnut aroma, more stuffy	93.17 ± 1.03	sweet and fresh; brisk	93.00 ± 0.71	93.27 ± 0.50 b
	S5	bright greenish yellow	94.17 ± 1.03	rich and lasting chestnut aroma	95.00 ± 0.00	sweet and fresh; brisk	93.33 ± 0.47	94.17 ± 0.39 ab
	S6	bright greenish yellow	95.00 ± 0.82	rich and lasting chestnut aroma	95.33 ± 0.47	sweet and fresh; brisk	93.50 ± 0.41	94.53 ± 0.50 a
	S7	bright greenish yellow	95.17 ± 0.62	chestnut aroma; approaching rich and lasting	94.67 ± 0.47	sweet and fresh; brisk	94.17 ± 0.85	94.57 ± 0.42 a
	S8	bright greenish yellow	95.33 ± 0.62	chestnut aroma; approaching rich and lasting	94.50 ± 0.41	fresh and heavy; brisk and sweet aftertaste	94.33 ± 0.94	94.60 ± 0.45 a
	S9	bright light greenish yellow	93.67 ± 0.47	chestnut aroma; approaching rich and lasting	94.17 ± 0.24	fresh and heavy; brisk and sweet aftertaste	93.33 ± 0.94	93.73 ± 0.41 ab
	CK	bright light greenish	93.50 ± 0.41	rich chestnut aroma	93.00 ± 0.82	fresh and heavy; brisk	93.17 ± 1.03	93.17 ± 0.65 bc
Black tea	S1	deep red; more bright	90.17 ± 0.62	rich, sweet aroma; a little dull odor	90.00 ± 0.82	heavy and mellow; sweet and more brisk; smooth	89.33 ± 1.25	89.77 ± 0.87 f
	S2	deep red; more bright	90.50 ± 0.71	rich, sweet aroma; a little dull odor	92.33 ± 0.47	heavy and mellow; sweet, more fresh, and brisk; smooth	90.17 ± 0.62	91.10 ± 0.37 e
	S3	deep red; approaching bright	92.17 ± 0.85	rich, sweet aroma; slightly dull odor	92.67 ± 0.47	heavy and mellow; sweet, more fresh, and brisk; smooth	91.67 ± 0.47	92.17 ± 0.29 d
	S4	deep red; approaching bright	93.00 ± 0.82	rich, sweet aroma; slightly dull odor	93.67 ± 1.18	fresh and sweet; approaching brisk	93.00 ± 0.71	93.27 ± 0.50 c
	S5	bright red	94.83 ± 0.24	rich, sweet aroma; slightly dull odor	94.17 ± 0.85	fresh and sweet; brisk	94.33 ± 0.47	94.37 ± 0.45 ab
	S6	bright red	95.00 ± 0.00	sweet aroma; rich and lasting	94.83 ± 0.94	fresh and sweet; brisk	94.33 ± 0.47	94.67 ± 0.50 a
	S7	bright orange-red	94.33 ± 0.24	sweet aroma; rich and lasting	94.17 ± 0.47	fresh and sweet; brisk	93.67 ± 0.47	94.00 ± 0.22 abc
	S8	bright orange-red	94.17 ± 0.47	sweet aroma; rich and lasting	93.83 ± 0.62	fresh and sweet; approaching brisk	93.33 ± 0.94	93.70 ± 0.49 abc
	S9	bright orange-red	93.67 ± 0.47	sweet aroma; rich and lasting	93.67 ± 0.47	fresh and sweet; approaching brisk	93.33 ± 0.94	93.53 ± 0.41 bc
	CK	bright orange-red	93.50 ± 0.41	sweet aroma; rich and lasting	94.00 ± 0.82	fresh and heavy; brisk	93.50 ± 0.71	93.7 ± 0.45 abc

Significant differences between the ten formula waters (including CK) at the same tea are indicated by different lowercase letters (*p* < 0.05).

**Table 3:** Contents of catechins and caffeine in green tea at different brewing times after brewing with tea brewing water 1 (TBW1) and CK.

Brewing Times	Water Sample	GC μg/mL	EGC μg/mL	C μg/mL	EC μg/mL	EGCG μg/mL	GCG μg/mL	ECG μg/mL	CG μg/mL	GA μg/mL	CAF μg/mL
1 min	CK	58.45 ± 18.61 Ae	48.41 ± 6.38 Af	21.27 ± 7.62 Ae	40.40 ± 12.28 Ae	108.35 ± 20.32 Ae	37.04 ± 2.57 Af	25.08 ± 3.60 Ae	28.27 ± 2.24 Af	8.79 ± 2.51 Ae	202.35 ± 17.19 Ae
	TBW1	63.76 ± 1.84 Af	54.93 ± 1.01 Af	23.94 ± 1.40 Af	42.10 ± 0.70 Af	114.14 ± 11.07 Af	37.07 ± 0.85 Af	26.12 ± 1.98 Af	29.49 ± 0.21 Af	12.03 ± 0.43 Af	209.38 ± 6.24 Af
2 min	CK	114.48 ± 12.89 Ad	121.47 ± 3.57 Ae	66.15 ± 9.71 Ad	96.47 ± 10.7 Ad	402.27 ± 92.59 Ad	93.52 ± 4.59 Ae	87.17 ± 22.19 Ad	66.65 ± 3.31 Ae	20.69 ± 2.89 Ad	483.21 ± 36.80 Ad
	TBW1	130.95 ± 1.69 Ae	118.64 ± 7.74 Ae	60.63 ± 2.85 Ae	92.71 ± 4.98 Ae	298.40 ± 38.51 Ae	83.90 ± 2.36 Be	64.79 ± 7.21 Ae	65.98 ± 2.24 Ae	24.28 ± 0.45 Ae	472.12 ± 11.05 Ae
3 min	CK	158.63 ± 11.54 Ac	173.36 ± 2.91 Ad	102.10 ± 15.66 Acd	137.85 ± 11.63 Ac	613.84 ± 76.22 Ac	142.26 ± 3.10 Ad	131.38 ± 18.27 Ac	97.19 ± 3.94 Ad	29.06 ± 3.74 Ac	672.46 ± 40.95 Ac
	TBW1	171.62 ± 1.58 Ad	164.54 ± 8.82 Ad	91.42 ± 3.60 Ad	132.76 ± 7.25 Ad	455.68 ± 45.63 Bd	127.82 ± 3.14 Bd	99.06 ± 8.86 Ad	95.60 ± 1.87 Ad	32.61 ± 0.70 Ad	655.23 ± 5.58 Ad
5 min	CK	191.05 ± 9.44 Bb	223.36 ± 1.51 Ac	133.74 ± 17.87 Abc	178.36 ± 11.67 Ab	842.46 ± 60.51 Ab	193.28 ± 5.56 Ac	179.97 ± 13.6 Ab	128.13 ± 3.72 Ac	36.34 ± 4.04 Ab	796.37 ± 38.64 Ab
	TBW1	210.70 ± 2.30 Ac	212.76 ± 9.28 Ac	121.34 ± 3.08 Ac	171.86 ± 6.75 Ac	605.90 ± 33.96 Bc	171.18 ± 2.78 Bc	131.57 ± 7.18 Bc	126.94 ± 1.55 Ac	40.32 ± 0.72 Ac	786.46 ± 0.51 Ac
7 min	CK	205.72 ± 8.34 Bab	248.74 ± 1.77 Ab	167.35 ± 30.28 Ab	199.3 ± 13.02 Aab	980.23 ± 56.44 Aa	234.13 ± 5.81 Ab	210.58 ± 12.77 Aa	148.36 ± 3.05 Ab	42.18 ± 4.10 Ab	856.67 ± 33.89 Aab
	TBW1	231.19 ± 2.17 Ab	239.36 ± 10.52 Ab	142.52 ± 2.28 Ab	199.35 ± 7.81 Ab	712.19 ± 34.22 Bb	208.81 ± 2.36 Bb	156.26 ± 7.15 Bb	148.22 ± 2.28 Ab	46.02 ± 0.45 Ab	855.64 ± 2.19 Ab
10 min	CK	214.96 ± 7.86 Ba	266.44 ± 4.53 Aa	210.33 ± 35.66 Aa	213.67 ± 14.18 Aa	1087.76 ± 51.28 Aa	271.31 ± 5.96 Aa	235.49 ± 11.89 Aa	164.42 ± 2.80 Aa	47.71 ± 4.00 Aa	887.27 ± 31.59 Aa
	TBW1	245.70 ± 2.99 Aa	258.57 ± 10.89 Aa	159.15 ± 3.09 Aa	220.91 ± 7.94 Aa	802.70 ± 36.7 Ba	244.28 ± 2.30 Ba	178.19 ± 7.38 Ba	166.54 ± 2.20 Aa	52.26 ± 0.53 Aa	893.47 ± 2.95 Aa

Note: Lowercase letters represent the significance analysis of different brewing times under the same water sample; the significance analysis of different water samples under the same brewing time is indicated using capital letters.

## Data Availability

The original contributions presented in this study are included in the article and Supplementary Material. Further inquiries can be directed to the corresponding authors.

## Supplementary Materials

The following supporting information can be downloaded at: https://www.mdpi.com/article/10.3390/foods15111958/s1, Figure S1: Pictures of 7 kinds of tea; Figure S2: Pictures of tea infusion after brewing 7 kinds of tea with formula water; Table S1: Sensory evaluation of tea after brewing with formula water; Table S2: Physicochemical properties of water with different concentrations of HCO_3_^−^; Table S3: Sensory evaluation of tea after brewing with water with different concentrations of HCO_3_^−^; Table S4: The content of catechins and caffeine for green tea and black tea after brewing with different concentrations of HCO_3_^−^; Table S5: The contents of catechins and caffeine in black tea at different brewing time after brewing with tea brewing water (TBW2) and CK; Table S6: The contents of catechins and caffeine in tea infusion after brewing with 4 types of water.
