# Supplementary material for: Effects of Bicarbonate Ions in Tea Brewing Water on Sensory Properties and Substances of Different Teas
Source: Foods. 2026 Jun 2;15(11):1958. doi: 10.3390/foods15111958 (PMC13256915; doi:10.3390/foods15111958)
Supplement: Supplementary file 1 [file foods-15-01958-s001.zip › foods-4039940-supplementary.pdf]

Supporting Information

Effects of Bicarbonate Ions in Tea Brewing Water on Sensory Properties and Substance of Different Teas

Wanjun Gao[1], Xing Liang[1], Lanying Li[1], Yu Yao[1], Bo Zhang[2], Yongjiang Hu[2], Fan Luo[1], Dongna Liu[1, *]

[1] Tea Research Institute, Tea Resources Utilization and Quality Testing Key Laboratory of Sichuan Province, Sichuan Academy of Agricultural Sciences, Chengdu, 610066, PR China

[2] Sichuan Yihuo Quanjiang Beverage Co., Ltd., Guangyuan, 628000, PR China

* Corresponding author

Figure captions

Figure S1 Pictures of 7 kinds of tea

Figure S2 Pictures of tea soup after brewing 7 kinds of tea with formula water

Table S1 Sensory evaluation of tea after brewing with formula water

Tea	Water	Soup color（20%）		Aroma（40%）		Taste（40%）		Total score
		Comment	Score	Comment	Score	Comment	Score	
white tea	S1	light yellow approach bright	89.50±0.71	clean and refrehing have tip aroma, lasting	88.67±1.25	sweet, approach fresh and brisk	90.00±0.82	89.37±0.73e
	S2	light yellow approach bright	90.17±0.24	clean and refrehing have tip aroma, lasting	89.67±0.85	sweet, approach fresh and brisk	90.17±0.62	89.97±0.54e
	S3	light yellow approach bright	91.83±0.24	tip aroma approach rich, lasting	90.33±0.24	sweet and fresh, approach brisk	91.33±0.47	91.03±0.26d
	S4	light yellow approach bright	90.83±0.62	tip aroma approach rich, lasting	91.50±0.41	sweet and fresh, brisk	92.33±0.24	91.70±0.37cd
	S5	bright apricot	92.67±0.24	tip aroma rich and lasting	92.17±0.24	sweet and fresh, brisk	92.83±0.24	92.53±0.21ab
	S6	bright apricot	92.83±0.24	tip aroma rich and lasting	92.50±0.41	sweet and fresh, brisk	93.00±0.41	92.77±0.05ab
	S7	bright apricot	92.67±0.62	tip aroma rich and lasting	93.17±0.24	sweet and fresh, brisk	93.50±0.41	93.20±0.37a
	S8	bright apricot	92.50±0.71	tip aroma rich and lasting	92.67±0.47	sweet and fresh, brisk	93.17±0.24	92.83±0.12ab
	S9	bright apricot	92.33±0.85	tip aroma rich and lasting	92.17±0.85	sweet and fresh, brisk	92.17±0.24	92.20±0.22bc
	CK	bright apricot	92.17±0.24	tip aroma approach rich, lasting	91.50±0.41	sweet and fresh, approach brisk	91.67±0.62	91.70±0.37cd

Table S1 (*continued*)

Tea	Water	Soup color（20%）		Aroma（40%）		Taste（40%）		Total score
		Comment	Score	Comment	Score	Comment	Score	
yellow tea	S1	apricot approach bright	89.83±0.24	tend aroma with fry bean aroma	88.50±0.41	heavy and mellow more brisk, have burnt and bitter taste	87.83±0.62	88.50±0.37g
	S2	yellow approach bright	90.67±0.47	tend aroma with fry bean aroma	89.83±0.62	heavy and mellow approach brisk, have burnt and bitter taste	90.33±0.47	90.20±0.33f
	S3	bright honey yellow	91.33±0.62	tend aroma with fry bean aroma	91.50±0.41	heavy and mellow approach brisk	91.83±0.24	91.60±0.24e
	S4	bright honey yellow	92.17±0.47	tend aroma with fry bean aroma	92.00±0.82	heavy and mellow, brisk	93.5±0.71	92.63±0.50de
	S5	bright honey yellow	92.67±0.62	tend aroma with sweet flowery aroma	94.17±0.85	heavy and mellow, brisk	94.5±0.71	94.00±0.73abc
	S6	bright honey yellow	94.83±0.85	tend aroma with sweet flowery aroma	95.00±0.71	heavy and mellow, sweet and brisk	95.33±0.94	95.10±0.70a
	S7	bright honey yellow	94.33±0.85	tend aroma with sweet flowery aroma	94.83±0.85	heavy and mellow, sweet and brisk	94.33±0.94	94.53±0.71ab
	S8	bright honey yellow	93.50±0.41	tend aroma with sweet flowery aroma	93.33±0.47	heavy and mellow, sweet and brisk	93.67±0.47	93.50±0.37bcd
	S9	bright honey yellow	93.00±0.82	tend aroma with sweet flowery aroma	93.00±0.82	heavy and mellow, sweet and brisk	93±0.82	93.00±0.71cd
	CK	bright honey yellow	93.50±0.71	tend aroma with sweet flowery aroma	94.50±0.71	heavy and mellow, sweet and brisk	93.33±0.47	93.83±0.45bc

Table S1 (*continued*)

Tea	Water	Soup color（20%）		Aroma（40%）		Taste（40%）		Total score
		Comment	Score	Comment	Score	Comment	Score	
Oolong tea	S1	bright yellowish green	89.00±0.82	clean and refreshing	90.17±0.24	strong approach mellow, smooth	89.00±0.82	89.47±0.52e
	S2	bright yellowish green	90.33±0.47	clean and refreshing	91.00±0.82	strong approach mellow, smooth	90.50±0.71	90.67±0.52d
	S3	bright light yellowish green	91.50±0.41	clean and refreshing show flowery aroma	92.33±0.47	heavy and mellow, smooth	91.33±0.94	91.77±0.58c
	S4	bright light yellowish green	92.67±0.47	rich clean and flowery aroma	92.83±0.62	heavy and mellow, smooth approach brisk	92.67±0.47	92.73±0.38b
	S5	bright light yellowish green	93.17±0.47	rich clean and flowery aroma	94.17±0.85	heavy and mellow, smooth approach brisk	92.83±0.24	93.43±0.41ab
	S6	bright light yellowish green	93.33±0.62	rich clean and flowery aroma	94.00±0.82	heavy and mellow, smooth and brisk	94.00±0.41	93.87±0.46a
	S7	bright light yellow	92.17±0.24	clean and refreshing show flowery aroma	94.17±0.24	heavy and mellow, smooth and brisk	94.17±0.62	93.77±0.17a
	S8	bright light yellow	91.50±0.41	clean and refreshing show flowery aroma	94.00±0.41	heavy and mellow, smooth and brisk	93.83±0.85	93.43±0.12ab
	S9	bright light yellow	91.17±0.24	clean and refreshing show flowery aroma	93.83±0.62	mellow approach strong, smooth and brisk	93.17±0.62	93.03±0.12ab
	CK	bright light yellow	92.50±0.71	clean and refreshing show flowery aroma	93.00±0.41	mellow approach strong, smooth and brisk	93.00±1.08	92.90±0.36b

Table S1 (*continued*)

Tea	Water	Soup color（20%）		Aroma（40%）		Taste（40%）		Total score
		Comment	Score	Comment	Score	Comment	Score	
Dark tea	S1	deep red more dull	88.67±0.94	aroma after aging rich and lasting	91.83±0.62	mellow and thick, sweet after taste, smooth	93.33±0.47	91.80±0.59ef
	S2	bright red rich	93.67±0.47	aroma after aging approach pure and normol, rich and lasting	93.50±0.41	mellow and thick, sweet after taste, smooth have stale flavor character	94.17±0.62	93.80±0.43bc
	S3	red rich a little dull	92.50±0.71	aroma after aging pure and normol, rich and lasting	94.00±0.82	mellow and thick, sweet after taste, smooth have stale flavor character	94.83±0.62	94.03±0.21b
	S4	bright red rich	94.67±0.47	aroma after aging pure and normol, rich and lasting	94.83±0.62	mellow and thick, sweet after taste, smooth have stale flavor character	95.33±0.47	95.00±0.28a
	S5	bright red rich	94.50±0.41	aroma after aging pure and normol, rich and lasting	93.67±0.47	mellow and thick, sweet after taste, smooth have stale flavor character	94.83±0.24	94.30±0.14ab
	S6	bright red	93.33±0.47	aroma after aging pure and normol, rich and lasting	93.00±0.82	mellow and thick, sweet after taste, have stale flavor character	93.00±0.82	93.07±0.66cd
	S7	bright red	93.00±0.82	aroma after aging pure and normol, approach rich and lasting	92.33±0.85	stale and mellow approach thick, sweet after taste	92.67±0.47	92.60±0.59de
	S8	red approach bright	92.17±0.85	aroma after aging pure and normol, approach rich	91.17±0.85	stale and mellow approach thick, sweet after taste	92.00±0.82	91.70±0.64ef
	S9	red more bright	91.67±0.47	aroma after aging pure and normol more lasting	90.67±0.47	stale and mellow more thick, sweet after taste	91.5±0.41	91.20±0.16fg
	CK	red more bright	91.17±0.24	aroma after aging pure and normol more lasting	90.50±0.41	stale and mellow more thick, sweet after taste	90.67±0.47	90.70±0.22g

Table S1 (*continued*)

Tea	Water	Soup color（20%）		Aroma（40%）		Taste（40%）		Total score
		Comment	Score	Comment	Score	Comment	Score	
scented tea	S1	yellow more bright	87.33±0.94	flowery aroma more fresh lovely, rich and more lasting	89.33±0.94	strong more fresh, more brisk, sweet after taste	88.67±0.94	88.67±0.19f
	S2	greenish yellow approach bright	89.67±0.47	flowery aroma approach fresh lovely, rich and approach lasting	90.67±0.47	strong approach fresh, approach brisk, sweet after taste	90.33±0.47	90.33±0.09e
	S3	bright greenish yellow	92.00±0.82	flowery aroma approach fresh lovely, rich and lasting	91.67±0.47	strong approach fresh, approach brisk, sweet after taste	91.67±0.24	91.73±0.34d
	S4	bright greenish yellow	93.00±0.82	flowery aroma approach fresh lovely, rich and lasting	92.83±0.62	strong approach fresh, brisk and sweet after taste	92.33±0.47	92.67±0.52c
	S5	bright greenish yellow	94.17±1.43	flowery aroma and fresh lovely, rich and lasting	93.50±0.41	strong approach fresh, brisk and sweet after taste	92.50±0.71	93.23±0.54bc
	S6	bright greenish yellow	93.67±0.85	flowery aroma and fresh lovely, rich and lasting	93.33±0.47	strong approach fresh, brisk and sweet after taste	92.67±0.62	93.13±0.29bc
	S7	bright light greenish yellow	93.83±0.62	flowery aroma and fresh lovely, rich and lasting	93.83±0.62	fresh and heavy, brisk and sweet after taste	93.00±0.82	93.50±0.45ab
	S8	bright light greenish yellow	93.17±0.62	flowery aroma and fresh lovely, rich and lasting	93.67±0.47	fresh and heavy, brisk and sweet after taste	93.33±0.94	93.43±0.21ab
	S9	bright light greenish yellow	93.00±0.82	flowery aroma and fresh lovely, rich and lasting	93.67±0.47	fresh and heavy, brisk and sweet after taste	93.33±0.94	93.40±0.16abc
	CK	bright light greenish yellow	94.50±0.41	flowery aroma and fresh lovely, rich and lasting	93.83±0.62	fresh and heavy, brisk and sweet after taste	94.17±0.24	94.10±0.41a

Table S2 Physicochemical properties of water with different concentrations of HCO_3^-

Sample	Concentration of HCO_3^-(mg/L)	Concentration of Na^+(mg/L)	pH	Conductivity(μS/cm)
SY1	120.82	44.69	8.15±0.01	200.0
SY2	40.27	14.90	7.86±0.01	70.2
SY3	21.97	8.12	7.72±0.02	38.0
SY4	15.10	5.59	7.63±0.01	26.6
SY5	11.50	4.26	7.51±0.01	20.8
SY6	7.80	2.88	7.43±0.01	13.6
SY7	5.89	2.18	7.23±0.01	8.7

Table S3 Sensory evaluation of tea after brewing with water with different concentrations of HCO_3^-

Tea	Water	Soup color（20%）		Aroma（40%）		Taste（40%）		Total score
		Comment	Score	Comment	Score	Comment	Score	
Green tea	SY1	yellowish green more bright	89.83±0.24	chestnut aroma with clean and refreshing, more weak	89.00±0.82	strong more mellow, more bitter	87.50±1.08	88.57±0.69d
	SY2	yellowish green more bright	91.83±0.85	chestnut aroma with clean and refreshing	92.33±0.47	heavy and mellow approach fresh and brisk, with bitter	89.83±0.24	91.23±0.42c
	SY3	yellowish green more bright	92.33±0.47	chestnut aroma with clean and refreshing	93.33±0.47	fresh and brisk, with bitter and astringent	92.33±0.62	92.73±0.50b
	SY4	bright yellowish green	94.00±0.82	chestnut aroma with clean and refreshing	94.17±0.62	fresh and brisk, with bitter and astringent	93.00±0.82	93.67±0.50ab
	SY5	bright yellowish green	94.33±0.62	chestnut aroma with clean and refreshing	94.00±0.82	fresh and brisk, with bitter and astringent	93.50±0.41	93.87±0.29a
	SY6	bright yellowish green	94.17±0.85	chestnut aroma with clean and refreshing	92.50±0.41	fresh and brisk, a little bitter and astringent	93.83±0.62	93.37±0.31ab
	SY7	bright yellowish green	93.33±0.62	chestnut aroma with clean and refreshing	92.17±0.24	fresh and brisk, a little bitter and astringent	93.17±0.62	92.80±0.22bc
	CK	bright yellowish green	93.17±0.62	chestnut aroma with clean and refreshing, more weak	90.50±0.41	fresh and brisk, a little bitter and astringent	91.50±0.41	91.43±0.12c

Table S3 (*continued*)

Tea	Water	Soup color（20%）		Aroma（40%）		Taste（40%）		Total score
		Comment	Score	Comment	Score	Comment	Score	
Black tea	SY1	red more bright	89.00±0.82	sweet aroma more rich	89.00±0.82	sweet and mellow	88.83±0.85	89.10±0.67e
	SY2	bright red	92.33±0.47	sweet aroma, rich and lasting	92.33±0.47	sweet and mellow, more fresh	90.67±1.03	91.80±0.49d
	SY3	bright red	93.67±0.94	sweet aroma, rich and lasting	93.67±0.94	fresh and sweet, mellow	93.83±0.85	93.80±0.59bc
	SY4	bright red	94.67±0.47	sweet aroma, rich and lasting	94.67±0.47	fresh and sweet, mellow	94.33±0.47	94.43±0.24ab
	SY5	bright red	93.67±0.47	sweet aroma, rich and lasting	93.67±0.47	fresh and sweet, mellow, a little sour	93.17±0.24	93.60±0.16bc
	SY6	bright red	92.83±0.85	sweet aroma, rich and lasting	92.83±0.85	fresh and sweet, mellow, a little sour	92.33±0.47	92.97±0.46c
	SY7	bright red	94.33±0.47	sweet aroma, rich and lasting	92.67±0.47	fresh and sweet, mellow, a little sour	91.67±0.94	92.60±0.43cd
	CK	red approach bright	91.17±0.62	sweet aroma more rich, lasting	91.17±0.62	sweet and mellow, approach fresh, a little sour	91.50±0.41	91.83±0.26d

Table S3 (*continued*)

Tea	Water	Soup color（20%）		Aroma（40%）		Taste（40%）		Total score
		Comment	Score	Comment	Score	Comment	Score	
white tea	SY1	bright orange	89.00±0.82	clean and refreshing have tip aroma, lasting	89.00±0.82	mellow and sweet after taste, smooth	90.17±0.24	89.47±0.57d
	SY2	bright deep apricot	90.50±0.41	clean and refreshing have tip aroma, lasting	90.67±0.47	mellow and sweet after taste, smooth	91.17±0.62	90.83±0.45c
	SY3	bright apricot	92.17±0.24	tip aroma approach rich, lasting	91.83±0.24	mellow and sweet after taste, approach fresh	92.17±0.24	92.03±0.12b
	SY4	bright apricot	92.83±0.62	tip aroma, rich and lasting	93.50±0.41	fresh and sweet, brisk	93.00±0.41	93.17±0.39a
	SY5	apricot approach bright	91.33±0.47	tip aroma, rich and lasting	93.83±0.62	fresh and sweet, brisk	93.50±0.41	93.20±0.43a
	SY6	bright apricot	92.83±0.24	tip aroma, rich and lasting	93.33±0.47	fresh and sweet, brisk	92.33±0.24	92.83±0.12a
	SY7	bright apricot	92.33±0.47	tip aroma, rich and lasting	92.5±0.41	fresh and sweet, approach brisk	91.33±0.47	92.00±0.33bc
	CK	bright apricot	91.67±0.47	tip aroma approach rich, lasting	91.67±0.62	sweet and fresh, approach brisk	90.67±0.47	91.27±0.09c

Table S3 (*continued*)

Tea	Water	Soup color（20%）		Aroma（40%）		Taste（40%）		Total score
		Comment	Score	Comment	Score	Comment	Score	
yellow tea	SY1	apricot approach bright	89.17±0.85	fry bean aroma, rich more high	89.17±1.03	heavy and mellow more brisk, have burnt and bitter taste	87.83±0.62	88.63±0.45d
	SY2	yellow approach bright	90.33±0.47	fry bean aroma, rich more high	90.50±1.08	heavy and mellow approach brisk, have burnt and bitter taste	89.5±0.41	90.07±0.41c
	SY3	bright honey yellow	91.17±0.62	fry bean aroma, rich approach high	91.67±0.47	heavy and mellow, sweet and brisk	90.83±0.62	91.23±0.33b
	SY4	bright honey yellow	92.00±0.82	fry bean aroma, rich and high	93.00±0.82	heavy and mellow, sweet and brisk	92.5±0.41	92.60±0.33a
	SY5	bright honey yellow	92.50±0.82	fry bean aroma, rich and high	93.33±0.85	heavy and mellow, sweet and brisk	91.83±0.24	92.57±0.25a
	SY6	bright honey yellow	92.17±0.62	fry bean aroma, rich and high	92.33±0.62	heavy and mellow, sweet and brisk	90.33±0.47	91.50±0.24b
	SY7	bright honey yellow	92.00±0.82	fry bean aroma, rich and high	92.00±0.82	heavy and mellow, sweet and brisk	90.50±0.41	91.40±0.16b
	CK	bright honey yellow	92.00±0.82	fry bean aroma, rich approach high	91.33±0.47	heavy and mellow, approach sweet and brisk	90.17±1.03	91.00±0.33b

Table S3 (*continued*)

Tea	Water	Soup color（20%）		Aroma（40%）		Taste（40%）		Total score
		Comment	Score	Comment	Score	Comment	Score	
oolong tea	SY1	bright yellowish green	89.17±0.62	clean and refreshing	90.33±0.47	heavy and mellow	87.83±0.62	89.10±0.51e
	SY2	yellowish green more bright	87.67±0.47	clean and refreshing	91.00±0.41	heavy and mellow	90.17±0.24	90.00±0.28d
	SY3	bright little yellowish green	92.33±0.47	clean and refreshing show flowery aroma	91.67±0.47	heavy and mellow, more sweet and brisk	91.17±0.24	91.60±0.28c
	SY4	bright little yellowish green	92.50±0.41	rich clean and flowery aroma	93.33±0.47	heavy and mellow, sweet and brisk	92.67±0.62	92.90±0.22a
	SY5	bright little yellowish green	93.33±0.24	rich clean and flowery aroma	92.67±0.47	heavy and mellow, sweet and brisk	93.33±0.47	93.07±0.05a
	SY6	bright little yellowish green	92.67±0.47	rich clean and flowery aroma	92.00±0.41	heavy and mellow, sweet and brisk	92.50±0.41	92.33±0.09b
	SY7	bright little yellowish green	92.50±0.41	clean and refreshing show flowery aroma	91.33±0.94	heavy and mellow, sweet and brisk	92.33±0.94	91.97±0.45cd
	CK	bright little yellowish green	92.17±0.24	clean and refreshing show flowery aroma	91.83±0.62	heavy and mellow, more sweet and brisk	90.83±0.85	91.50±0.22c

Table S3 (*continued*)

Tea	Water	Soup color（20%）		Aroma（40%）		Taste（40%）		Total score
		Comment	Score	Comment	Score	Comment	Score	
dark tea	SY1	deep red more bright	88.67±0.94	aroma after aging pure and normol, rich and more lasting	91.33±0.47	mellow and thick, smooth	92.67±0.94	91.33±0.38c
	SY2	deep red approach bright	91.00±0.82	aroma after aging pure and normol, rich and lasting	94.00±0.41	mellow and thick, sweet after taste, smooth have aged flavour	94.33±0.47	93.53±0.09a
	SY3	bright red rich	93.33±0.94	aroma after aging pure and normol, rich and lasting	94.83±0.24	mellow and thick, sweet after taste, smooth have aged flavour	93.67±0.94	94.07±0.41a
	SY4	bright red rich	95.00±0.82	aroma after aging pure and normol, rich and lasting	94.17±0.62	mellow and thick, sweet after taste, have aged flavour	93.00±0.82	93.87±0.52a
	SY5	bright red rich	94.17±0.62	aroma after aging pure and normol, rich and lasting	92.83±0.62	mellow and thick, sweet after taste, have aged flavour	91.83±0.85	92.70±0.08b
	SY6	bright red rich	93.67±0.94	aroma after aging pure and normol, rich and lasting	92.67±0.47	mellow and thick, sweet after taste, have aged flavour	90.33±0.47	91.93±0.19c
	SY7	red rich approach bright	93.67±0.94	aroma after aging pure and normol, rich and lasting	92.67±0.47	mellow and thick, sweet after taste	90.33±0.47	91.93±0.19de
	CK	red rich approach bright	92.00±0.82	aroma after aging pure and normol, more rich and lasting	91.00±0.82	stale and mellow, more thick	89.33±0.94	90.53±0.47d

Table S3 (*continued*)

Tea	Water	Soup color（20%）		Aroma（40%）		Taste（40%）		Total score
		Comment	Score	Comment	Score	Comment	Score	
scented tea	SY1	greenish yellow more bright	89.17±0.85	rich flowery aroma more fresh lovely, a little dull odour	87.33±0.94	strong more fresh, more brisk, sweet after taste	86.17±0.85	87.23±0.88f
	SY2	greenish yellow more bright	90.00±0.82	rich flowery aroma more fresh lovely, a little dull odour	90.17±0.85	strong approach fresh, approach brisk, sweet after taste	89.50±0.41	89.87±0.25e
	SY3	greenish yellow approach bright	91.33±0.47	flowery aroma and fresh lovely, rich and more lasting	92.83±0.85	strong approach fresh, approach brisk, sweet after taste	90.83±0.62	91.73±0.34d
	SY4	bright greenish yellow	92.17±0.24	flowery aroma and fresh lovely, rich and lasting	94.17±1.31	fresh and heavy, brisk and sweet after taste, have flowery	93.50±0.41	93.50±0.64bc
	SY5	bright greenish yellow	93.50±0.71	flowery aroma and fresh lovely, rich and lasting	94.00±0.82	fresh and heavy, brisk and sweet after taste, have flowery	95.17±0.24	94.37±0.21ab
	SY6	bright light greenish yellow	93.67±0.47	flowery aroma and fresh lovely, more rich and lasting	94.50±0.41	fresh and heavy, brisk and sweet after taste, have flowery	94.33±0.47	94.27±0.25ab
	SY7	bright light greenish yellow	93.33±0.47	flowery aroma and fresh lovely, more rich and lasting	93.00±0.82	strong, approach fresh and brisk, sweet after taste, have flowery	93.50±0.41	93.27±0.34b
	CK	bright light greenish yellow	93.17±0.24	flowery aroma and fresh lovely, more rich and lasting	92.67±0.24	strong, approach fresh and brisk, sweet after taste	92.50±0.41	92.70±0.14cd

Table S4 The content of catechins and caffeine for green tea and black tea after brewing with different concentrations of HCO_3^-

Tea	Sample	GC μg/mL	EGC μg/mL	C μg/mL	EC μg/mL	EGCG μg/mL	GCG μg/mL	ECG μg/mL	CG μg/mL	GA μg/mL	CAF μg/mL
Green tea	SY1	101.62±2.61b	120.99±4.53c	12.88±0.46b	95.52±7.53a	383.23±3.73a	63.20±1.03a	79.47±1.04a	58.77±2.09a	11.40±0.30b	398.94±9.54ab
	SY2	96.87±1.24b	123.63±0.53bc	47.03±30.19a	95.35±3.50a	382.14±1.20a	61.71±0.65ab	79.29±0.39a	59.21±1.68a	11.06±0.25b	393.97±3.60ab
	SY3	95.86±4.16b	124.46±1.58bc	66.35±6.54a	93.41±3.79a	376.95±17.80a	60.56±3.01ab	77.12±3.84a	59.56±1.86a	10.81±0.50b	396.74±14.60ab
	SY4	99.80±5.46b	119.98±10.00c	61.87±4.80a	90.00±5.51a	373.51±32.92a	59.51±1.82ab	76.24±5.84a	56.06±3.86a	10.62±0.87b	380.41±19.72b
	SY5	106.75±12.08b	132.45±6.10ab	61.43±4.35a	93.51±2.96a	403.14±1.22a	59.96±2.15ab	82.44±1.02a	58.11±3.45a	11.25±0.30b	394.13±12.40b
	SY6	102.15±1.47b	117.77±6.32c	65.70±0.77a	95.04±2.50a	382.30±48.84a	59.59±1.62ab	78.83±11.08a	59.15±1.17a	11.01±0.31b	407.92±2.30a
	SY7	125.87±7.75a	137.45±2.31a	60.65±4.03a	95.94±1.52a	376.91±21.94a	58.55±3.36b	76.30±5.13a	58.85±1.33a	11.95±2.37b	398.49±3.13ab
	CK	132.92±7.86a	126.88±8.94bc	63.50±3.20a	96.04±9.91a	403.68±25.23a	58.50±3.10b	74.85±14.48a	59.89±2.51a	15.66±4.08a	407.18±16.20a
Black tea	SY1	77.01±1.32c	——	56.18±4.46a	——	——	——	25.78±0.74b	33.48±0.65a	68.00±1.27a	476.38±9.31b
	SY2	73.96±7.05c	——	48.71±5.20b	——	——	——	25.67±0.26b	32.75±1.57a	67.97±2.51a	492.60±10.66ab
	SY3	80.75±13.22bc	——	33.70±5.50c	——	——	——	25.83±0.64b	33.59±0.05a	67.42±1.03a	491.23±11.08ab
	SY4	89.64±2.08ab	——	25.57±1.06d	——	——	——	25.59±0.54b	32.78±0.84a	68.61±2.18a	498.47±11.37a
	SY5	94.19±1.11a	——	23.16±0.22d	——	——	——	25.47±0.32b	32.98±0.76a	67.69±1.02a	503.16±6.82a
	SY6	92.22±0.79a	——	24.48±1.04d	——	——	——	26.39±0.29b	33.11±1.48a	67.13±0.91a	498.68±9.08a
	SY7	94.14±2.09a	——	24.01±2.58d	——	——	——	26.20±1.39b	32.54±2.11a	66.02±1.31a	508.59±13.05a
	CK	81.27±2.50bc	——	24.93±1.40d	——	——	——	28.65±1.02a	29.75±1.36b	62.80±2.00b	425.56±11.68c

Table S5 The contents of catechins and caffeine in black tea at different brewing time after brewing with tea brewing water (TBW2) and CK

Brewing times	Water sample	GC μg/ml	C μg/ml	CG μg/ml	GA μg/ml	CAF μg/ml
1 min	CK	35.59±7.95Ae	8.3±0.24Ad	15.29±0.5Af	35.42±1.33Af	217.88±8.86Af
	TBW2	35.09±1.39Ae	7.58±0.19Bd	0±0Bd	30.79±1.31Bf	230.82±10.11Ae
2 min	CK	83.62±6.27Ad	24.01±0.62Ac	34.23±0.16Ae	76.4±0.35Ae	507.07±5.14Ae
	TBW2	77.58±2.64Ad	21.63±0.95Bc	19.97±0.7Bc	66.5±1.83Be	522.84±13.36Ad
3 min	CK	119.08±5.37Ac	39.65±1.6Ab	51.26±0.77Ad	101.93±0.84Ad	705.25±13.76Ad
	TBW2	113.07±1.67Ac	36.26±0.5Bb	38.72±0.31Bb	91.64±2.12Bd	746.72±21.99Ac
5 min	CK	152.34±3.4Ab	60.26±1.17Aa	69.9±1.24Ac	124.17±1.88Ac	847.18±19.91Ac
	TBW2	141.64±2.1Bb	53.04±0.15Ba	56.72±0.14Ba	110.12±2.47Bc	884.58±25.54Ab
7 min	CK	171.38±3.16Aa	60.26±1.17Aa	83.2±1.33Ab	135.27±2.2Ab	918.43±21.05Ab
	TBW2	158.25±2.1Ba	53.04±0.15Ba	56.72±0.14Ba	120.88±2.53Bb	956.81±27.18Aa
10 min	CK	171.38±3.16Aa	60.26±1.17Aa	95.02±1.49Aa	144.53±2.54Aa	959.1±21.01Aa
	TBW2	158.25±2.1Ba	53.04±0.15Ba	56.72±0.14Ba	129.93±2.56Ba	994.58±27.33Aa

Note: Lowercase letters represent the significance analysis of different brewing times under the same water sample; The significance analysis of different water samples under the same brewing time using capital letters.

Table S6 The contents of catechins and caffeine in tea soup after brewing with 4 types of water

Tea	Water sample	GC μg/ml	EGC μg/ml	C μg/ml	EC μg/ml	EGCG μg/ml	GCG μg/ml	ECG μg/ml	CG μg/ml	GA μg/ml	CAF μg/ml
Green tea	PW	96.76±3.9a	126.61±6.66ab	61.64±6.04ab	62.62±54.46c	409.07±27.69a	67.27±4.63a	77.71±5.12a	57.94±1.81a	10.19±0.52a	395.04±9.19a
	TW	105.34±12.2a	121.30±7.6b	50.12±1.02c	218.98±41.74b	430.18±54.88a	72.28±3.04a	81.70±6.92a	56.53±5.80a	10.88±0.24a	399.54±18.62a
	MW	96.02±2.05a	124.82±4.56ab	57.10±1.87bc	272.43±9.48a	422.01±25.58a	69.83±2.58a	82.74±5.19a	60.15±2.50a	10.56±0.22a	392.19±6.22a
	TBW1	100.03±1.53a	132.41±3.94a	66.56±4.52a	282.32±5.64a	439.46±44.95a	70.78±4.44a	85.28±7.5a	62.90±3.49a	10.18±0.42a	411.9±18.04a

Table S6 (*continued*)

Tea	Water sample	GC μg/ml	C μg/ml	CG μg/ml	GA μg/ml	CAF μg/ml
Black tea						
	PW	75.35±3.02a	19.34±5.80a	29.08±1.34a	61.5±1.94a	435.67±15.57a
	TW	72.31±1.49ab	20.73±0.64a	27.49±0.53a	61.73±2.03a	406.52±8.57b
	MW	74.68±1.05a	22.79±2.65a	29.87±1.61a	64.02±3.14a	426.71±15.18ab
	TBW2	70.39±1.86b	21.85±4.41a	28.51±1.95a	61.72±2.21a	407.03±15.65b

Figure S1

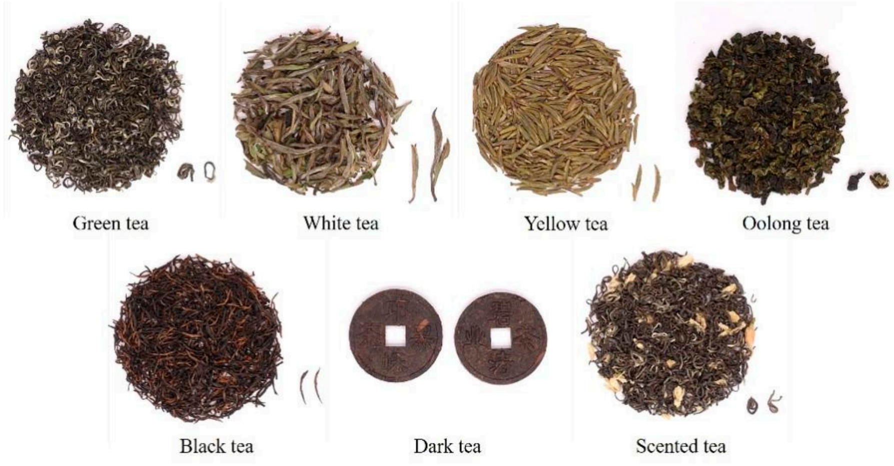

Figure S1 Pictures of 7 kinds of tea

Figure S2

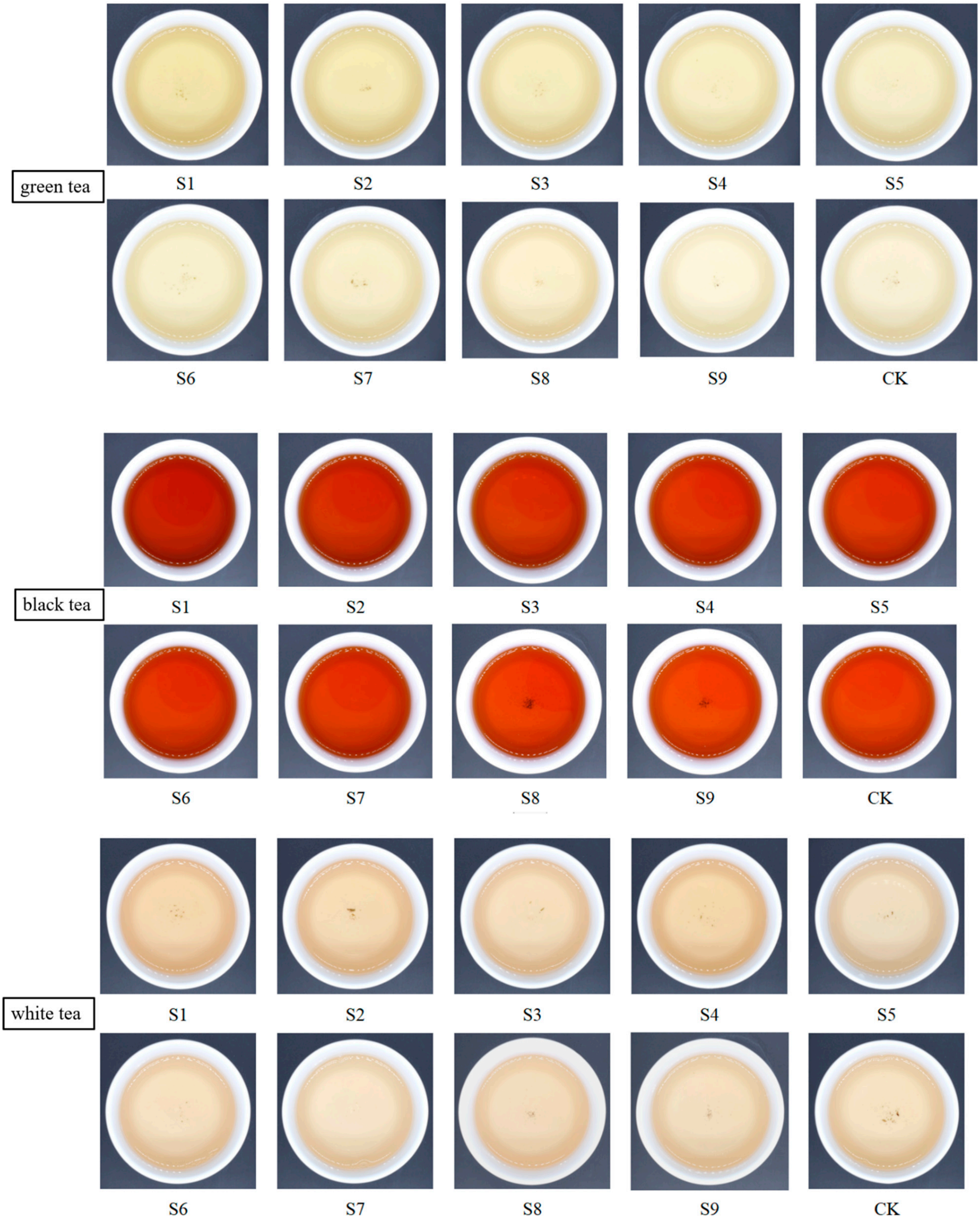
green tea
S1
S2
S3
S4
S5
S6
S7
S8
S9
CK
black tea
S1
S2
S3
S4
S5
S6
S7
S8
S9
CK
white tea
S1
S2
S3
S4
S5
S6
S7
S8
S9
CK

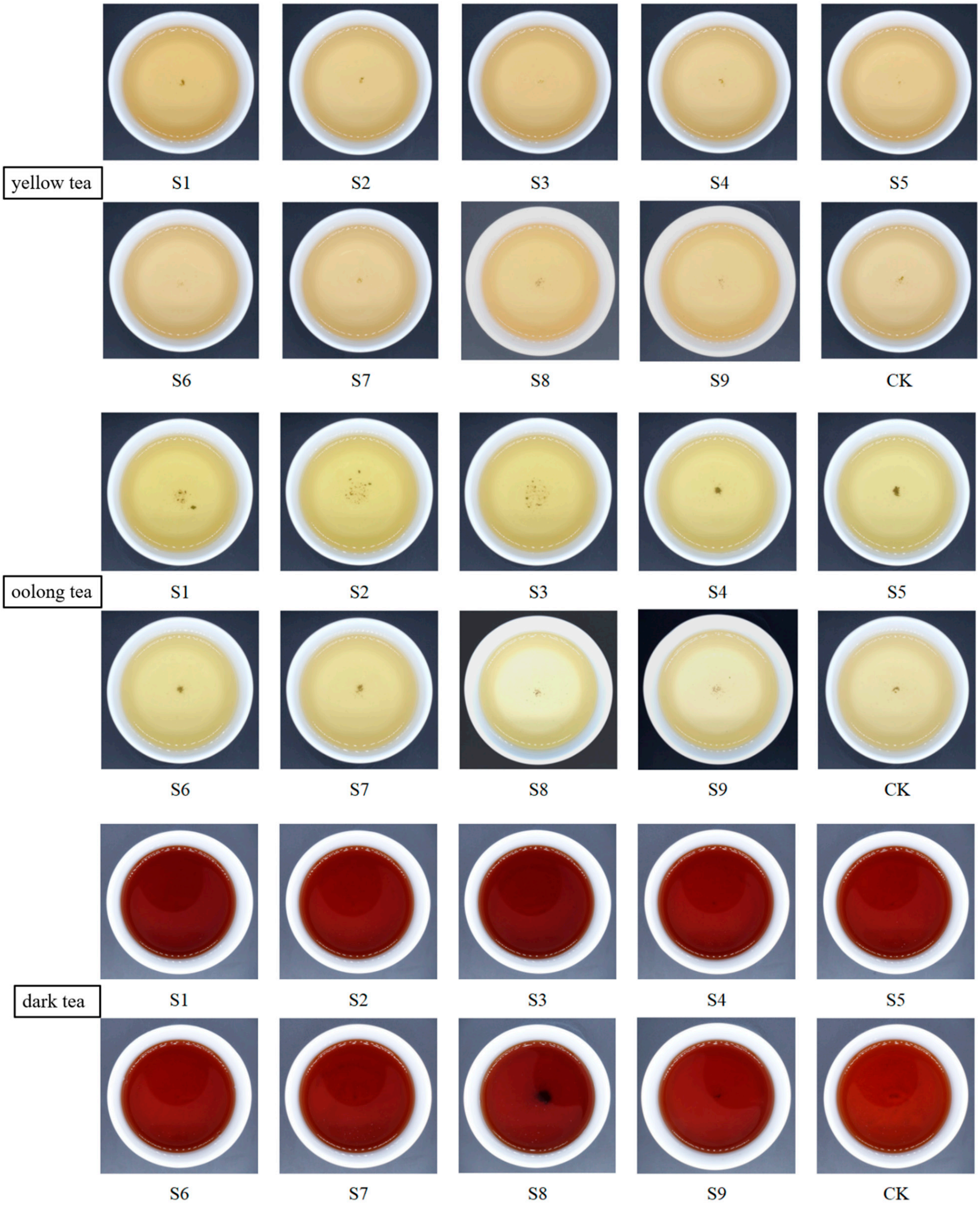
yellow tea
S1
S2
S3
S4
S5
S6
S7
S8
S9
CK
oolong tea
S1
S2
S3
S4
S5
S6
S7
S8
S9
CK
dark tea
S1
S2
S3
S4
S5
S6
S7
S8
S9
CK

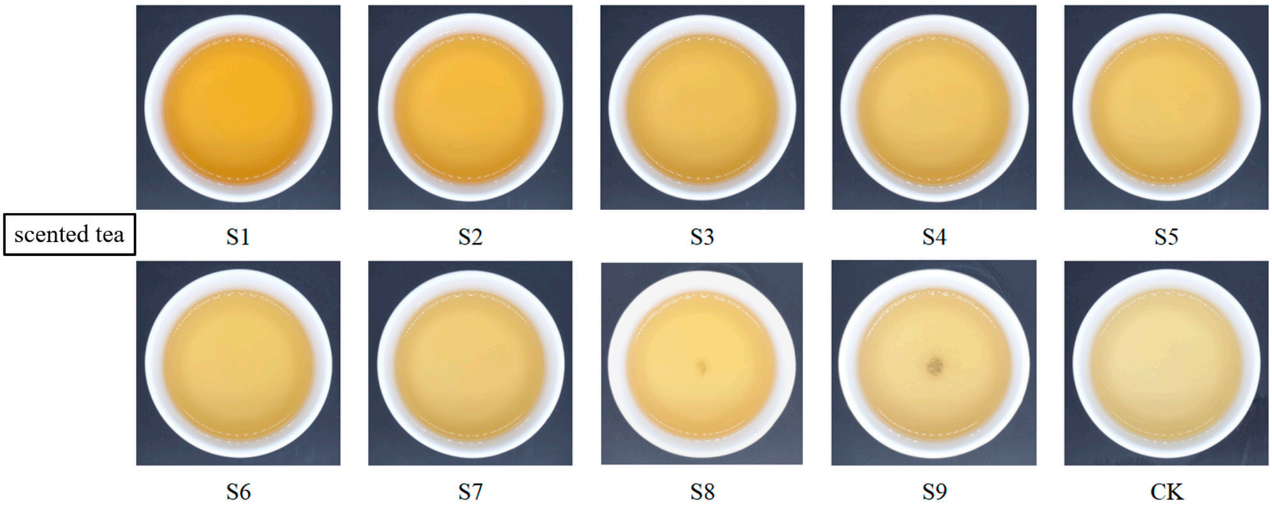

Figure S2 Pictures of tea soup after brewing 7 kinds of tea with formula water
